# Bromidotetra­kis­(2-isopropyl-1*H*-imidazole-κ*N* ^3^)copper(II) bromide

**DOI:** 10.1107/S1600536811035215

**Published:** 2011-09-14

**Authors:** Sylwia Godlewska, Joanna Socha, Katarzyna Baranowska, Anna Dołęga

**Affiliations:** a Department of Inorganic Chemistry, Faculty of Chemistry, Gdansk University of Technology, 11/12 G. Narutowicz St, 80952 – PL Gdańsk, Poland

## Abstract

The Cu^II^ atom in the title salt, [CuBr(C_6_H_10_N_2_)_4_]Br, is coordinated in a square-pyramidal geometry by four imidazole N atoms and one bromide anion that is located at the apex of the pyramid. The cations and the anions form a two-dimensional network parallel to (001) through N—H⋯Br hydrogen bonds.

## Related literature

For similar compounds, see: Hossaini Sadr *et al.* (2004 ▶); Li *et al.* (2007 ▶); Liu *et al.* (2007 ▶).
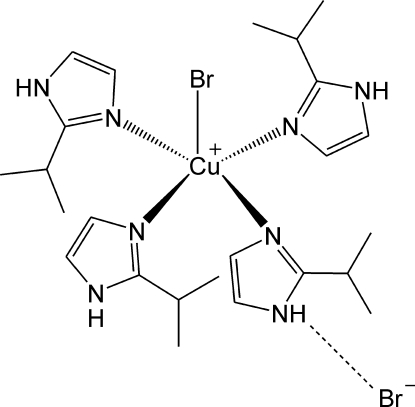


## Experimental

### 

#### Crystal data

[CuBr(C_6_H_10_N_2_)_4_]Br
*M* *_r_* = 664
Monoclinic, 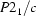

*a* = 10.7094 (7) Å
*b* = 19.9917 (6) Å
*c* = 16.7885 (19) Å
β = 121.552 (7)°
*V* = 3063.0 (4) Å^3^
*Z* = 4
Mo *K*α radiation
μ = 3.35 mm^−1^
*T* = 120 K
0.41 × 0.25 × 0.23 mm

#### Data collection

Oxford Diffraction Xcalibur Sapphire2 diffractometer
Absorption correction: multi-scan (*CrysAlis PRO*; Oxford Diffraction, 2010 ▶) *T*_min_ = 0.628, *T*_max_ = 1
11133 measured reflections
5710 independent reflections
4597 reflections with *I* > 2σ(*I*)
*R*_int_ = 0.017

#### Refinement

*R*[*F* ^2^ > 2σ(*F* ^2^)] = 0.033
*wR*(*F* ^2^) = 0.092
*S* = 1.05
5710 reflections
324 parameters
H-atom parameters constrained
Δρ_max_ = 1.86 e Å^−3^
Δρ_min_ = −1.04 e Å^−3^

### 

Data collection: *CrysAlis PRO* (Oxford Diffraction, 2010 ▶); cell refinement: *CrysAlis PRO*; data reduction: *CrysAlis PRO*; program(s) used to solve structure: *SHELXS97* (Sheldrick, 2008 ▶); program(s) used to refine structure: *SHELXL97* (Sheldrick, 2008 ▶); molecular graphics: *ORTEP-3* (Farrugia, 1997 ▶); software used to prepare material for publication: *WinGX* (Farrugia, 1999 ▶).

## Supplementary Material

Crystal structure: contains datablock(s) I, global. DOI: 10.1107/S1600536811035215/ng5221sup1.cif

Structure factors: contains datablock(s) I. DOI: 10.1107/S1600536811035215/ng5221Isup2.hkl

Additional supplementary materials:  crystallographic information; 3D view; checkCIF report

## Figures and Tables

**Table 1:** Hydrogen-bond geometry (Å, °)

*D*—H⋯*A*	*D*—H	H⋯*A*	*D*⋯*A*	*D*—H⋯*A*
N2—H2*A*⋯Br2	0.88	2.48	3.358 (2)	175
N4—H4*A*⋯Br2^i^	0.88	2.48	3.342 (2)	167
N6—H6*D*⋯Br2^ii^	0.88	2.53	3.351 (2)	155
N8—H8*A*⋯Br2^iii^	0.88	2.49	3.362 (2)	169

Symmetry codes: (i) 
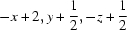
; (ii) 
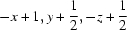
; (iii) 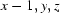.

**Fig. 1.**
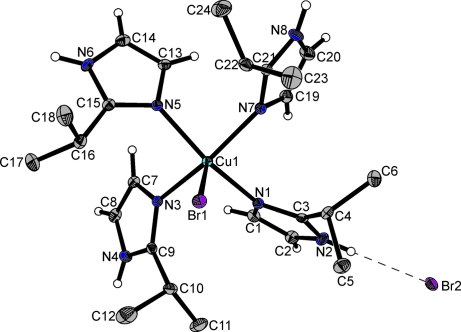
A view of (I), showing the atom-labelling scheme. Displacement ellipsoids are drawn at the 30% probability level. H atoms of isopropyl groups have been omitted. Hydrogen bonds indicated with dashed lines.

**Fig. 2.**
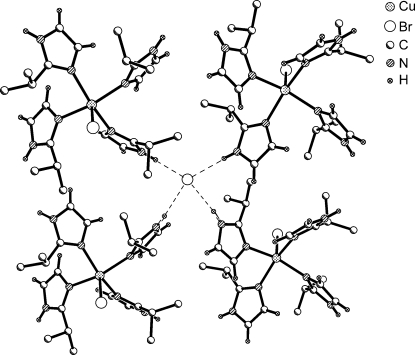
The packing of (I) along the c axis. H atoms of isopropyl groups have been omitted. Hydrogen bonds indicated with dashed lines.

## supplementary crystallographic information

### Comment

Title compound was synthesized as a substrate for further synthesis of mixed ligand copper complexes.

The structure of the complex ion in (I) is similar to those described earlier (Hossaini Sadr *et al.* (2004); Li *et al.* (2007); Liu *et al.* (2007)). However, Cu1—Br1 bond in (I) [2.6608 (6) Å] is significantly shorter compared to the Cu1—Br1 bond found in bromotetrakis(1*H*-imidazole-κ*N*^3^)copper(II) bromide [2.755 (1) Å] (Hossaini Sadr *et al.* (2004)). The steric hindrance introduced with the isopropyl group causes the rotation of the planes of imidazole rings and the hydrogen bond formed by Br1 in (1*H*-imidazole-κ*N*^3^)copper(II) bromide is no longer present in (I). This obviously results in the strengthening and shortening of Cu1—Br1. The two-dimensional hydrogen bonding network in (I) consists of four NH···Br hydrogen bonds formed by Br2.

The structure of (I) is shown in Fig. 1 and crystal packing diagram is presented in Fig.2.

### Experimental

Compound (I) was prepared by the reaction of 2-isopropylimidazole (0.496 g, 4.5 mmol) with CuBr_2_ (0.223 g, 1 mmol) in methanol and slow evaporation of solvent from the reaction solution.

### Refinement

All C–H hydrogen atoms were refined as riding on carbon atoms with methyl C–H = 0.98 Å, methine C–H = 1 Å, aromatic C–H = 0.95 Å and *U*_iso_(H)=1.2 *U*_eq_(C)for aromatic and methine CH and 1.5*U*_eq_(C) for methyl groups. The final difference Fourier map had a peak/hole in the vicinity of the Br atoms.

### Crystal data

**Table:**

[CuBr(C_6_H_10_N_2_)_4_]Br	*F*(000) = 1356
*M**_r_* = 664	*D*_x_ = 1.44 Mg m^−^^3^
Monoclinic, *P*2_1_/*c*	Mo *K*α radiation, λ = 0.71073 Å
Hall symbol: -P 2ybc	Cell parameters from 4456 reflections
*a* = 10.7094 (7) Å	θ = 2.5–30.0°
*b* = 19.9917 (6) Å	µ = 3.35 mm^−^^1^
*c* = 16.7885 (19) Å	*T* = 120 K
β = 121.552 (7)°	Prism, blue
*V* = 3063.0 (4) Å^3^	0.41 × 0.25 × 0.23 mm
*Z* = 4	

### Data collection

**Table:**

Oxford Diffraction Xcalibur Sapphire2 diffractometer	5710 independent reflections
graphite	4597 reflections with *I* > 2σ(*I*)
Detector resolution: 8.1883 pixels mm^-1^	*R*_int_ = 0.017
ω scans	θ_max_ = 25.5°, θ_min_ = 2.5°
Absorption correction: multi-scan (*CrysAlis PRO*; Oxford Diffraction, 2010)	*h* = −12→11
*T*_min_ = 0.628, *T*_max_ = 1	*k* = −14→24
11133 measured reflections	*l* = −18→20

### Refinement

**Table:**

Refinement on *F*^2^	Primary atom site location: structure-invariant direct methods
Least-squares matrix: full	Secondary atom site location: difference Fourier map
*R*[*F*^2^ > 2σ(*F*^2^)] = 0.033	Hydrogen site location: inferred from neighbouring sites
*wR*(*F*^2^) = 0.092	H-atom parameters constrained
*S* = 1.05	*w* = 1/[σ^2^(*F*_o_^2^) + (0.0602*P*)^2^] where *P* = (*F*_o_^2^ + 2*F*_c_^2^)/3
5710 reflections	(Δ/σ)_max_ = 0.001
324 parameters	Δρ_max_ = 1.86 e Å^−^^3^
0 restraints	Δρ_min_ = −1.04 e Å^−^^3^

### Special details

**Table:**

Geometry. All s.u.'s (except the s.u. in the dihedral angle between two l.s. planes) are estimated using the full covariance matrix. The cell s.u.'s are taken into account individually in the estimation of s.u.'s in distances, angles and torsion angles; correlations between s.u.'s in cell parameters are only used when they are defined by crystal symmetry. An approximate (isotropic) treatment of cell s.u.'s is used for estimating s.u.'s involving l.s. planes.
Refinement. Refinement of *F*^2^ against ALL reflections. The weighted *R*-factor *wR* and goodness of fit *S* are based on *F*^2^, conventional *R*-factors *R* are based on *F*, with *F* set to zero for negative *F*^2^. The threshold expression of *F*^2^ > 2σ(*F*^2^) is used only for calculating *R*-factors(gt) *etc*. and is not relevant to the choice of reflections for refinement. *R*-factors based on *F*^2^ are statistically about twice as large as those based on *F*, and *R*- factors based on ALL data will be even larger.

### Fractional atomic coordinates and isotropic or equivalent isotropic displacement parameters (Å^2^)

**Table:**

	*x*	*y*	*z*	*U*_iso_*/*U*_eq_	
Br1	0.31171 (3)	0.779079 (15)	0.001639 (19)	0.02020 (10)	
Cu1	0.45146 (4)	0.766296 (16)	0.18655 (2)	0.01437 (10)	
N1	0.6390 (3)	0.71702 (11)	0.21910 (16)	0.0171 (5)	
N2	0.8002 (3)	0.64385 (13)	0.23226 (17)	0.0223 (6)	
H2A	0.8388	0.6095	0.22	0.027*	
N3	0.5719 (3)	0.85202 (11)	0.22788 (16)	0.0161 (5)	
N4	0.7488 (3)	0.92415 (12)	0.26694 (17)	0.0215 (6)	
H4A	0.8168	0.9478	0.2654	0.026*	
N5	0.3114 (2)	0.81177 (11)	0.21723 (16)	0.0157 (5)	
N6	0.1974 (3)	0.88848 (12)	0.24787 (17)	0.0219 (6)	
H6D	0.1516	0.926	0.2444	0.026*	
N7	0.3763 (3)	0.67785 (11)	0.20488 (16)	0.0176 (5)	
N8	0.2343 (3)	0.60629 (12)	0.21834 (18)	0.0247 (6)	
H8A	0.1554	0.5839	0.206	0.03*	
C1	0.7738 (3)	0.73116 (15)	0.2985 (2)	0.0209 (7)	
H1	0.7924	0.7669	0.3406	0.025*	
C2	0.8743 (3)	0.68637 (15)	0.3069 (2)	0.0233 (7)	
H2	0.9752	0.6847	0.3544	0.028*	
C3	0.6587 (3)	0.66308 (14)	0.1806 (2)	0.0179 (6)	
C4	0.5460 (3)	0.62848 (14)	0.0936 (2)	0.0194 (6)	
H4	0.4525	0.6542	0.0673	0.023*	
C5	0.5900 (4)	0.62837 (17)	0.0204 (2)	0.0294 (8)	
H5A	0.6099	0.6743	0.0098	0.044*	
H5B	0.5099	0.6097	−0.0382	0.044*	
H5C	0.6782	0.6011	0.0428	0.044*	
C6	0.5164 (4)	0.55681 (15)	0.1124 (2)	0.0303 (8)	
H6A	0.6051	0.5298	0.1354	0.046*	
H6B	0.4365	0.5373	0.0544	0.046*	
H6C	0.4885	0.5576	0.1595	0.046*	
C7	0.6050 (3)	0.88407 (14)	0.3097 (2)	0.0194 (6)	
H7	0.5584	0.8759	0.3438	0.023*	
C8	0.7131 (3)	0.92847 (15)	0.3338 (2)	0.0229 (7)	
H8	0.7562	0.9571	0.3867	0.028*	
C9	0.6617 (3)	0.87732 (14)	0.2031 (2)	0.0174 (6)	
C10	0.6682 (3)	0.86072 (15)	0.1187 (2)	0.0220 (7)	
H10	0.6014	0.8219	0.0865	0.026*	
C11	0.8215 (4)	0.84085 (19)	0.1435 (3)	0.0378 (9)	
H11A	0.8889	0.8783	0.1749	0.057*	
H11B	0.8203	0.8294	0.0864	0.057*	
H11C	0.8543	0.802	0.1853	0.057*	
C12	0.6135 (4)	0.9195 (2)	0.0515 (3)	0.0472 (11)	
H12A	0.5132	0.9307	0.0342	0.071*	
H12B	0.6146	0.9076	−0.0048	0.071*	
H12C	0.6774	0.9582	0.0817	0.071*	
C13	0.2988 (3)	0.79106 (15)	0.2919 (2)	0.0196 (6)	
H13	0.3342	0.7499	0.3244	0.023*	
C14	0.2289 (3)	0.83811 (15)	0.3109 (2)	0.0231 (7)	
H14	0.2059	0.8368	0.3584	0.028*	
C15	0.2480 (3)	0.87125 (14)	0.1918 (2)	0.0177 (6)	
C16	0.2259 (3)	0.91264 (15)	0.1117 (2)	0.0217 (7)	
H16	0.2855	0.8924	0.0878	0.026*	
C17	0.2765 (4)	0.98551 (15)	0.1391 (2)	0.0305 (8)	
H17A	0.2169	1.0072	0.1604	0.046*	
H17B	0.2651	1.0096	0.0848	0.046*	
H17C	0.3797	0.9862	0.1896	0.046*	
C18	0.0635 (4)	0.90979 (17)	0.0320 (2)	0.0328 (8)	
H18A	0.0343	0.8631	0.0144	0.049*	
H18B	0.0506	0.9345	−0.0222	0.049*	
H18C	0.0024	0.93	0.0532	0.049*	
C19	0.4603 (3)	0.64040 (14)	0.2861 (2)	0.0225 (7)	
H19	0.5627	0.6452	0.3285	0.027*	
C20	0.3729 (4)	0.59637 (16)	0.2946 (2)	0.0273 (7)	
H20	0.4012	0.5648	0.3434	0.033*	
C21	0.2391 (3)	0.65596 (14)	0.1655 (2)	0.0184 (6)	
C22	0.1103 (3)	0.67838 (15)	0.0751 (2)	0.0223 (7)	
H22	0.1356	0.7223	0.0585	0.027*	
C23	0.0812 (4)	0.62825 (19)	−0.0015 (2)	0.0395 (9)	
H23A	0.17	0.6229	−0.0041	0.059*	
H23B	0.0013	0.6447	−0.0619	0.059*	
H23C	0.0536	0.585	0.0123	0.059*	
C24	−0.0242 (4)	0.68883 (19)	0.0820 (3)	0.0389 (9)	
H24A	−0.054	0.646	0.0955	0.058*	
H24B	−0.1044	0.7066	0.0227	0.058*	
H24C	−0.0011	0.7206	0.1324	0.058*	
Br2	0.96167 (4)	0.511235 (15)	0.20034 (3)	0.03505 (12)	

### Atomic displacement parameters (Å^2^)

**Table:**

	*U*^11^	*U*^22^	*U*^33^	*U*^12^	*U*^13^	*U*^23^
Br1	0.02145 (17)	0.02243 (16)	0.01668 (16)	−0.00041 (12)	0.00995 (13)	−0.00114 (12)
Cu1	0.01376 (19)	0.01431 (18)	0.01683 (19)	−0.00066 (14)	0.00923 (16)	−0.00082 (14)
N1	0.0161 (13)	0.0162 (12)	0.0198 (13)	0.0008 (10)	0.0100 (11)	0.0018 (11)
N2	0.0202 (14)	0.0239 (13)	0.0251 (14)	0.0077 (11)	0.0134 (12)	0.0037 (11)
N3	0.0166 (13)	0.0150 (11)	0.0185 (12)	−0.0001 (10)	0.0105 (11)	−0.0006 (10)
N4	0.0186 (13)	0.0196 (13)	0.0272 (14)	−0.0056 (11)	0.0126 (12)	−0.0028 (11)
N5	0.0147 (12)	0.0163 (12)	0.0173 (12)	−0.0005 (10)	0.0092 (11)	−0.0004 (10)
N6	0.0195 (14)	0.0232 (13)	0.0267 (14)	0.0048 (11)	0.0146 (12)	−0.0015 (12)
N7	0.0159 (13)	0.0158 (12)	0.0213 (13)	−0.0006 (10)	0.0099 (11)	−0.0008 (11)
N8	0.0226 (14)	0.0224 (13)	0.0306 (15)	−0.0056 (12)	0.0150 (13)	0.0033 (12)
C1	0.0190 (16)	0.0221 (15)	0.0180 (15)	−0.0021 (13)	0.0072 (13)	0.0001 (13)
C2	0.0159 (16)	0.0271 (16)	0.0214 (15)	0.0014 (13)	0.0059 (13)	0.0038 (14)
C3	0.0191 (16)	0.0183 (14)	0.0217 (15)	0.0013 (12)	0.0144 (13)	0.0052 (13)
C4	0.0203 (16)	0.0205 (15)	0.0203 (15)	0.0027 (13)	0.0125 (13)	−0.0015 (13)
C5	0.0328 (19)	0.0355 (19)	0.0232 (17)	0.0018 (16)	0.0170 (16)	0.0001 (15)
C6	0.036 (2)	0.0236 (16)	0.0334 (18)	−0.0031 (15)	0.0194 (17)	−0.0055 (15)
C7	0.0193 (16)	0.0206 (15)	0.0193 (15)	0.0007 (13)	0.0107 (13)	−0.0015 (13)
C8	0.0195 (16)	0.0241 (16)	0.0203 (16)	−0.0013 (13)	0.0071 (14)	−0.0078 (13)
C9	0.0152 (15)	0.0146 (14)	0.0216 (15)	−0.0002 (12)	0.0091 (13)	0.0000 (12)
C10	0.0230 (16)	0.0237 (15)	0.0236 (16)	−0.0039 (13)	0.0151 (14)	−0.0014 (13)
C11	0.038 (2)	0.048 (2)	0.041 (2)	0.0064 (18)	0.0295 (18)	0.0013 (18)
C12	0.052 (3)	0.066 (3)	0.030 (2)	0.022 (2)	0.027 (2)	0.018 (2)
C13	0.0194 (16)	0.0228 (15)	0.0194 (15)	0.0000 (13)	0.0121 (13)	0.0015 (13)
C14	0.0223 (16)	0.0285 (16)	0.0216 (15)	−0.0029 (14)	0.0136 (14)	−0.0033 (14)
C15	0.0129 (14)	0.0197 (15)	0.0184 (15)	−0.0025 (12)	0.0068 (13)	−0.0043 (12)
C16	0.0212 (16)	0.0219 (15)	0.0216 (15)	0.0047 (13)	0.0109 (14)	0.0013 (13)
C17	0.033 (2)	0.0225 (17)	0.0296 (18)	0.0012 (15)	0.0117 (16)	0.0066 (14)
C18	0.0311 (19)	0.0294 (17)	0.0248 (17)	0.0028 (15)	0.0056 (16)	0.0034 (15)
C19	0.0202 (16)	0.0223 (15)	0.0236 (15)	0.0034 (13)	0.0106 (14)	0.0060 (14)
C20	0.0262 (18)	0.0272 (17)	0.0256 (17)	0.0012 (14)	0.0115 (15)	0.0094 (15)
C21	0.0201 (16)	0.0158 (14)	0.0234 (15)	−0.0011 (12)	0.0141 (14)	−0.0008 (13)
C22	0.0182 (16)	0.0210 (15)	0.0267 (16)	−0.0027 (13)	0.0111 (14)	0.0018 (14)
C23	0.028 (2)	0.045 (2)	0.0281 (19)	−0.0014 (17)	0.0028 (16)	−0.0066 (17)
C24	0.0246 (19)	0.047 (2)	0.045 (2)	0.0101 (17)	0.0181 (18)	0.0131 (19)
Br2	0.0299 (2)	0.01770 (17)	0.0730 (3)	0.00417 (13)	0.0376 (2)	0.00779 (16)

### Geometric parameters (Å, °)

**Table:**

Br1—Cu1	2.6608 (6)	C7—H7	0.95
Cu1—N7	2.032 (2)	C8—H8	0.95
Cu1—N3	2.036 (2)	C9—C10	1.492 (4)
Cu1—N5	2.037 (2)	C10—C12	1.519 (5)
Cu1—N1	2.038 (2)	C10—C11	1.521 (4)
N1—C3	1.330 (4)	C10—H10	1
N1—C1	1.388 (4)	C11—H11A	0.98
N2—C3	1.350 (4)	C11—H11B	0.98
N2—C2	1.371 (4)	C11—H11C	0.98
N2—H2A	0.88	C12—H12A	0.98
N3—C9	1.332 (4)	C12—H12B	0.98
N3—C7	1.384 (3)	C12—H12C	0.98
N4—C9	1.360 (4)	C13—C14	1.340 (4)
N4—C8	1.364 (4)	C13—H13	0.95
N4—H4A	0.88	C14—H14	0.95
N5—C15	1.324 (4)	C15—C16	1.489 (4)
N5—C13	1.392 (4)	C16—C17	1.539 (4)
N6—C15	1.354 (4)	C16—C18	1.544 (4)
N6—C14	1.369 (4)	C16—H16	1
N6—H6D	0.88	C17—H17A	0.98
N7—C21	1.330 (4)	C17—H17B	0.98
N7—C19	1.394 (4)	C17—H17C	0.98
N8—C21	1.350 (4)	C18—H18A	0.98
N8—C20	1.375 (4)	C18—H18B	0.98
N8—H8A	0.88	C18—H18C	0.98
C1—C2	1.350 (4)	C19—C20	1.346 (4)
C1—H1	0.95	C19—H19	0.95
C2—H2	0.95	C20—H20	0.95
C3—C4	1.490 (4)	C21—C22	1.489 (4)
C4—C5	1.529 (4)	C22—C24	1.520 (4)
C4—C6	1.536 (4)	C22—C23	1.528 (5)
C4—H4	1	C22—H22	1
C5—H5A	0.98	C23—H23A	0.98
C5—H5B	0.98	C23—H23B	0.98
C5—H5C	0.98	C23—H23C	0.98
C6—H6A	0.98	C24—H24A	0.98
C6—H6B	0.98	C24—H24B	0.98
C6—H6C	0.98	C24—H24C	0.98
C7—C8	1.342 (4)		
			
N7—Cu1—N3	155.70 (9)	C9—C10—C11	112.2 (3)
N7—Cu1—N5	87.00 (9)	C12—C10—C11	110.3 (3)
N3—Cu1—N5	87.54 (9)	C9—C10—H10	108.1
N7—Cu1—N1	87.23 (9)	C12—C10—H10	108.1
N3—Cu1—N1	87.47 (9)	C11—C10—H10	108.1
N5—Cu1—N1	154.22 (9)	C10—C11—H11A	109.5
N7—Cu1—Br1	103.58 (7)	C10—C11—H11B	109.5
N3—Cu1—Br1	100.72 (7)	H11A—C11—H11B	109.5
N5—Cu1—Br1	102.30 (7)	C10—C11—H11C	109.5
N1—Cu1—Br1	103.47 (7)	H11A—C11—H11C	109.5
C3—N1—C1	106.4 (2)	H11B—C11—H11C	109.5
C3—N1—Cu1	130.2 (2)	C10—C12—H12A	109.5
C1—N1—Cu1	122.86 (19)	C10—C12—H12B	109.5
C3—N2—C2	109.2 (2)	H12A—C12—H12B	109.5
C3—N2—H2A	125.4	C10—C12—H12C	109.5
C2—N2—H2A	125.4	H12A—C12—H12C	109.5
C9—N3—C7	106.4 (2)	H12B—C12—H12C	109.5
C9—N3—Cu1	130.20 (19)	C14—C13—N5	109.6 (3)
C7—N3—Cu1	121.06 (18)	C14—C13—H13	125.2
C9—N4—C8	108.7 (2)	N5—C13—H13	125.2
C9—N4—H4A	125.7	C13—C14—N6	106.0 (3)
C8—N4—H4A	125.7	C13—C14—H14	127
C15—N5—C13	106.4 (2)	N6—C14—H14	127
C15—N5—Cu1	129.80 (19)	N5—C15—N6	109.2 (3)
C13—N5—Cu1	121.74 (19)	N5—C15—C16	127.1 (3)
C15—N6—C14	108.9 (2)	N6—C15—C16	123.7 (3)
C15—N6—H6D	125.6	C15—C16—C17	113.0 (3)
C14—N6—H6D	125.6	C15—C16—C18	109.7 (3)
C21—N7—C19	106.8 (2)	C17—C16—C18	110.8 (3)
C21—N7—Cu1	129.27 (19)	C15—C16—H16	107.7
C19—N7—Cu1	120.74 (19)	C17—C16—H16	107.7
C21—N8—C20	108.9 (2)	C18—C16—H16	107.7
C21—N8—H8A	125.5	C16—C17—H17A	109.5
C20—N8—H8A	125.5	C16—C17—H17B	109.5
C2—C1—N1	109.7 (3)	H17A—C17—H17B	109.5
C2—C1—H1	125.2	C16—C17—H17C	109.5
N1—C1—H1	125.2	H17A—C17—H17C	109.5
C1—C2—N2	105.5 (3)	H17B—C17—H17C	109.5
C1—C2—H2	127.3	C16—C18—H18A	109.5
N2—C2—H2	127.3	C16—C18—H18B	109.5
N1—C3—N2	109.2 (3)	H18A—C18—H18B	109.5
N1—C3—C4	126.9 (3)	C16—C18—H18C	109.5
N2—C3—C4	124.0 (3)	H18A—C18—H18C	109.5
C3—C4—C5	110.9 (3)	H18B—C18—H18C	109.5
C3—C4—C6	112.4 (3)	C20—C19—N7	109.0 (3)
C5—C4—C6	110.3 (3)	C20—C19—H19	125.5
C3—C4—H4	107.7	N7—C19—H19	125.5
C5—C4—H4	107.7	C19—C20—N8	106.2 (3)
C6—C4—H4	107.7	C19—C20—H20	126.9
C4—C5—H5A	109.5	N8—C20—H20	126.9
C4—C5—H5B	109.5	N7—C21—N8	109.1 (3)
H5A—C5—H5B	109.5	N7—C21—C22	126.9 (3)
C4—C5—H5C	109.5	N8—C21—C22	123.9 (3)
H5A—C5—H5C	109.5	C21—C22—C24	111.9 (3)
H5B—C5—H5C	109.5	C21—C22—C23	109.4 (3)
C4—C6—H6A	109.5	C24—C22—C23	111.7 (3)
C4—C6—H6B	109.5	C21—C22—H22	107.9
H6A—C6—H6B	109.5	C24—C22—H22	107.9
C4—C6—H6C	109.5	C23—C22—H22	107.9
H6A—C6—H6C	109.5	C22—C23—H23A	109.5
H6B—C6—H6C	109.5	C22—C23—H23B	109.5
C8—C7—N3	109.7 (3)	H23A—C23—H23B	109.5
C8—C7—H7	125.1	C22—C23—H23C	109.5
N3—C7—H7	125.1	H23A—C23—H23C	109.5
C7—C8—N4	106.2 (3)	H23B—C23—H23C	109.5
C7—C8—H8	126.9	C22—C24—H24A	109.5
N4—C8—H8	126.9	C22—C24—H24B	109.5
N3—C9—N4	108.9 (2)	H24A—C24—H24B	109.5
N3—C9—C10	127.8 (3)	C22—C24—H24C	109.5
N4—C9—C10	123.3 (3)	H24A—C24—H24C	109.5
C9—C10—C12	110.0 (3)	H24B—C24—H24C	109.5

### Hydrogen-bond geometry (Å, °)

**Table:**

*D*—H···*A*	*D*—H	H···*A*	*D*···*A*	*D*—H···*A*
N2—H2A···Br2	0.88	2.48	3.358 (2)	175.
N4—H4A···Br2^i^	0.88	2.48	3.342 (2)	167.
N6—H6D···Br2^ii^	0.88	2.53	3.351 (2)	155.
N8—H8A···Br2^iii^	0.88	2.49	3.362 (2)	169.

Symmetry codes: (i) −*x*+2, *y*+1/2, −*z*+1/2; (ii) −*x*+1, *y*+1/2, −*z*+1/2; (iii) *x*−1, *y*, *z*.
